# *In vitro* Evaluation of the Nematicidal Efficacy of Quercetin on Adult *Toxocara canis*

**DOI:** 10.1007/s11686-025-01026-x

**Published:** 2025-04-23

**Authors:** Rasha A. Elmahy, Nahla A. Radwan

**Affiliations:** Department of Zoology, Tanta University, Tanta, 31527 Egypt

**Keywords:** *Toxocara*, Quercetin, SEM, Histopathology, Oxidative enzymes

## Abstract

**Purpose:**

*Toxocara canis* is a globally distributed zoonotic parasite found in dogs' intestines, leading to various pathological damages, particularly to the intestinal flora. The larval stage causes human toxocariasis, especially in children, and may result in neurological disorders and blindness. Quercetin is a flavonoid with strong secondary metabolites and possesses medicinal advantages and antiparasitic qualities.

**Methods:**

The assay involved  four groups, each of  10 young adult *T. canis*; Group I was incubated in concentrations of an ethanolic extract of quercetin, Group II in albendazole (0.2 mM/ml) (+ve control), Group III in RPMI 1640 medium with ethanol (control), and Group IV in RPMI 1640 medium only (-ve control). The potential action of quercetin against adult *T. canis* in vitro was detected using scanning electron microscopy, histological investigations, and enzyme analysis.

**Results:**

SEM declared that exposure to LC90 of quercetin caused body shrinkage, cuticle and caudal papillae swelling, and disfigurement and erosion of cuticular annulations. Compared to albendazole's effect on the treated worm's body wall, results showed that quercetin generates oxidative stress and has an extensive and variable effect on *T. canis* organs, including the body wall, the gut, and the genitalia.

**Conclusion:**

Quercetin may set the stage for a new class of medications with remarkable potential for treating parasitic nematodes in dogs and could be extended to humans. This is the first time to employ a comprehensive study illuminating the potential action of quercetin against adult *Toxocara canis* in vitro.

**Supplementary Information:**

The online version contains supplementary material available at 10.1007/s11686-025-01026-x.

## Introduction

The dog roundworm *Toxocara canis* (Werner 1782) is the causative agent of human toxocariasis. As dogs are among our most popular pets, this zoonotic disease spreads globally. Children are the most vulnerable to infection by ingestion of embryonated *T. canis* eggs, which can be found in soil tainted with dog faeces. As larvae hatch in the stomach, they penetrate the mucosal epithelium and remain in the tissue phase, where they are developmentally stopped. Despite not growing or differentiating in humans, they continue to migrate and have an active metabolism [25], giving rise to visceral larva migrans (VLM), in which the major organs are affected, ocular larva migrans (OLM) when it affects the eye and can lead to unilateral blindness [22], and neural larva migrans (NLM) when it impacts the central nervous system [47].

In Egypt, the risk of contracting zoonotic parasite infection [5] has risen significantly due to the increase in dog breeders and stray dogs roaming freely in public spaces in recent years. According to certain research findings, the infection rate of *Toxocara canis* in dogs may vary from 10% to over 50% across the nation [1, 2, 30, 36]. Larvae are frequently spread through the placenta or the mother's milk, accounting for the higher occurrence in puppies. Furthermore, Egypt's hot and humid climate facilitates a quicker spread of *Toxocara* infection.

The most widely prescribed drug for treating human toxocariasis is the benzimidazole carbamate group [34]. However, because of their extremely low solubility, these medications have a low bioavailability in the tissues, necessitating the administration of relatively high doses over extended periods of time [15]. Furthermore, using these medications to treat other helminths may result in drug resistance [12]. Therefore, new drugs or alternatives for the treatment of human toxocariasis are urgently needed.

Within the group of bioflavonoids, quercetin (3,3',4',5,7-pentahydroxyflavone) is a significant compound that cannot be produced in the human body and is found in over twenty plant materials and has anti-inflammatory, antihypertensive, vasodilator, anti-obesity, antihypercholesterolemic, and antiatherosclerotic properties [40, 48]. When combined with fish oil, quercetin has been shown to have neuroprotective properties in the brains of rats and has been documented to exhibit protective properties against neurodegenerative illnesses [14, 18].

As documented in some studies, dogs may benefit from quercetin's anti-inflammatory and antioxidant properties, particularly when it comes to treating ailments like inflammation, arthritis, and allergies [3, 4]. It serves as an adjuvant treatment for intestinal diseases that may lead to compromised intestinal barrier integrity or disrupted microbiome [29]. It can lessen allergic responses and itching by stabilizing mast cells and inhibiting the release of histamine [6]. Its antioxidant qualities may also protect against oxidative stress and improve general cellular health.

Recently, there has been a push to harness quercetin's antioxidant properties as an antiparasitic focus. Most reports have focused on assessing the in vitro potentialities of *Leishmania*, *Trypanosoma*, and *Plasmodium* which are responsible for the majority of mortality and morbidity [39], where the action of quercetin was linked to elevated production of reactive oxygen species (ROS) and mitochondrial dysfunction [42]. According to other research, quercetin causes oxidative stress in the nervous system of the ruminant intestinal nematode *Haemonchus contortus*, which primarily alters metabolism and is likely linked to the worms' eventual physical harm and death [13].

Moreover, quercetin has the ability to significantly alter the helminth tegumental architecture and functions; additionally, it can directly affect or modify the metabolism of worms by interacting in a dose-dependent manner with enzymes or signalling molecules and controlling the RNA activity of several worm genes differently [11].

In the present study, our main objective was to evaluate the *in vitro* therapeutic potential of quercetin for adult *T. canis*. Our investigation also demonstrated, for the first time, the tissue morphology and histopathology in *T. canis* caused by exposure to quercetin. Our results revealed that quercetin causes oxidative stress, which kills adult worms by causing tissue damage.

## Materials and Methods

### *Toxocara canis*

*Toxocara canis* were collected from the intestines of five adult stray dogs (two females and three males) from the rural areas under the supervision of the Animal Health Research Institute, Ministry of Agriculture and Land Reclamation, Menofeyah  Governorate, Egypt. Euthanisation was performed by carbon monoxide inhalation. Dogs were carefully dissected, and the alimentary tracts were retrieved and transferred in phosphate buffered saline solution (PBS) at pH 7.4 to the laboratory. Young adults of *T. canis* were collected, washed in PBS, and identified under the microscope according to Skrjabin *et al*. [45].

## Albendazole

Pharmaceutical grade of PHR1281/albendazole, from Sigma-Aldrich Company, Saint Louis, MO, United States.

## Quercetin

Quercetin Dihydrate 98%, Oxford Health Company Ltd., UK was obtained by Biovision, Egypt Company, Egypt.

## Preparation of the Ethanolic Extract of Quercetin

A stock solution was prepared at a concentration of 6.6 mM/mL of a hydroalcoholic solution (60% ethanol and 40% water). Different concentrations were assessed and diluted using RPMI-1640 cultivation medium with l-glutamine (Roswell Park Memorial Institute-1640, Lot No. C4127) as follows: 0.25, 0.5, 0.75, 1, 1.25 and 1.5 mM/ml.

## *In vitro* Assay

### Assessment of Paralysis and Mortality Rate

Experiments were performed in a laminar flow cabinet (Nuaire NU 437-400E) under normal gas phase at 37 °C. The assay was conducted in four groups, each of ten young adults (5 males and 5 females); Group I received concentrations of quercetin ethanolic extract (0.25, 0.5, 0.75, 1, 1.25, and 1.5 mM/ml), Group II enclosed in albendazole (0.2 mM/ml) (+ve control), Group III incubated in RPMI 1640 medium with 2.19ml (44%) ethanol (control group), and Group  IV cultivated in the RPMI 1640 medium only (-ve control). The viability of the adult was checked by the mechanical shock using a needle and the heat stimuli; 50 °C for 10–15 seconds [13]. The percentages of paralysis and mortality were worked out from an average of three replicas. The observations were taken at regular time intervals: 2, 4, 6, 8, 10, 12, and 14 hours.

By deducting the dead worms in the control group from the total number of dead worms in the experimental group, the actual number of dead worms in each concentration of quercetin is determined. The percentage of adult mortality was calculated using the formula:

$${\text{Mortality rate }} = \frac{{{\text{Mean no}}.{\text{ of live worms in control group }} - {\text{Mean no}}.{\text{ of live worms in treated group}}}}{{ {\text{Mean no}}.{\text{ of live worms in control group}}}} \times {100}$$

The average and the standard deviation of mortality were calculated for all experimental concentrations at all exposure times.

The half lethal concentration (LC50) and the sub-lethal concentration (LC90) of ethanolic quercetin extract were determined at 8 hours of exposure according to Srinivasan [46]. Dose-response curve was conducted between the logarithm of the concentration on the x-axis and percentage mortality on the y-axis. The percentage of mortality was converted to a probit units using the following formula:

$${\text{P}} = {\text{a}} + {\text{b log}}\left( {\text{C}} \right)$$

where: P = Probit value corresponding to a specific mortality percentage; C = Concentration; a and b = Regression coefficients.

The concentration C that yields a probit value of 50% (for LC50) and 90% (for LC90) mortality was calculated.

For the histopathological analysis, antioxidant enzymes determination, and scanning electron microscopic inspection, three groups each of 10 young adults were prepared: group I, treated with LC90 of quercetin, group II, treated with LC90 of albendazole (0.2 mM/ml) [13], and group III, cultivated in RPMI-1640 with ethanol (control group). All the experiments were assessed under the normal gas phase at 37 °C for eight hours.

## Histopathological Analysis

Cultivated worms were cut into sections measuring one centimetre each; the anterior, middle, and posterior sections were separated, fixed in 10% formalin, transferred to an ascending series of ethyl alcohol, and immersed in paraffin. Formalin-fixed paraffin-embedded blocks were cut into 5 μm sections using a microtome. Hematoxylin and eosin (H&E) were used to stain the sections. Histopathological changes in the body wall and muscle layers were observed and photographed using a light microscope [49].

## Scanning Electron Microscopic Inspection

Adult worms were fixed in 70% ethyl alcohol, dehydrated, and carbon-dioxide critical point dried before being adjusted to aluminium stubs and coated with gold palladium in a gold sputter coating apparatus [7]. The specimens were viewed and photographed using a JSM-IT200Jeol (Japan) scanning electron microscope.

## Antioxidant Enzyme Activity Determination

The method described by Kar and Mishra [19] was used to extract total protein. Cultivated worms from all examined groups were washed in PBS, homogenized (10% wt/vol) in an ice-cold 0.1 M Tris HCl buffer (pH 7.4), centrifuged for 10 min at 3000 rpm, and the resulting supernatant was kept at − 20°C until used.

Superoxide dismutase (SOD) activity was determined using ELISA kits (Cat No. CSB-E08555r). The principle is based on assessing the prevention of reduction of p-nitro tetrazolium blue (NBT). Superoxide anion radicals are produced by xanthine and xanthine oxidase, and they quantitatively react with 2-(4-iodophenyl)-3-(4-nitrophenol)-5-phenyltetrazolium chloride to produce a red formazan dye. SOD turns the superoxide radical into oxygen, which stops the process [26].

Catalase (CAT) activities in each sample were measured using ELISA kits (Cat No. MBS2600683). The catalase reaction that breaks down hydrogen peroxide (H_2_O_2_) is the basis for the test principle where the excess H_2_O_2_ can form complexation with ammonium molybdate to produce a light-yellow solution. The concentration of H_2_O_2_ can be determined by measuring the solution's adsorption at 450 nm, which indirectly indicates the CAT activities [8].

Glutathione peroxidase (GSH-Px) activity was measured using ELISA kits (Cat No. CSB-E12146r) based on variations in the NADPH level. Hydrogen peroxide and cumene hydroperoxide (Sigma, St. Louis, Mo.) served as substrates; these were reduced by GPX with glutathione acting as the reducing agent [33].

All data were measured using a spectrophotometer. The enzyme units (U per millilitre; specific activity) were calculated as the change in absorbance per millilitre.

## Statistical Analysis

Data were presented as means and standard deviations of the independent experiments (experiments were triplicated). The LC50 and LC90 values for worm mortality were calculated using probit analysis. Two-way ANOVA test was established to understand the dependence of worm mortality on the tested concentrations of quercetin, the exposure times, and both factors. The statistical analysis of the data was conducted using SPSS.

## Results

### Paralysis and Mortality Assay (Fig. 1)

**Fig. 1:**
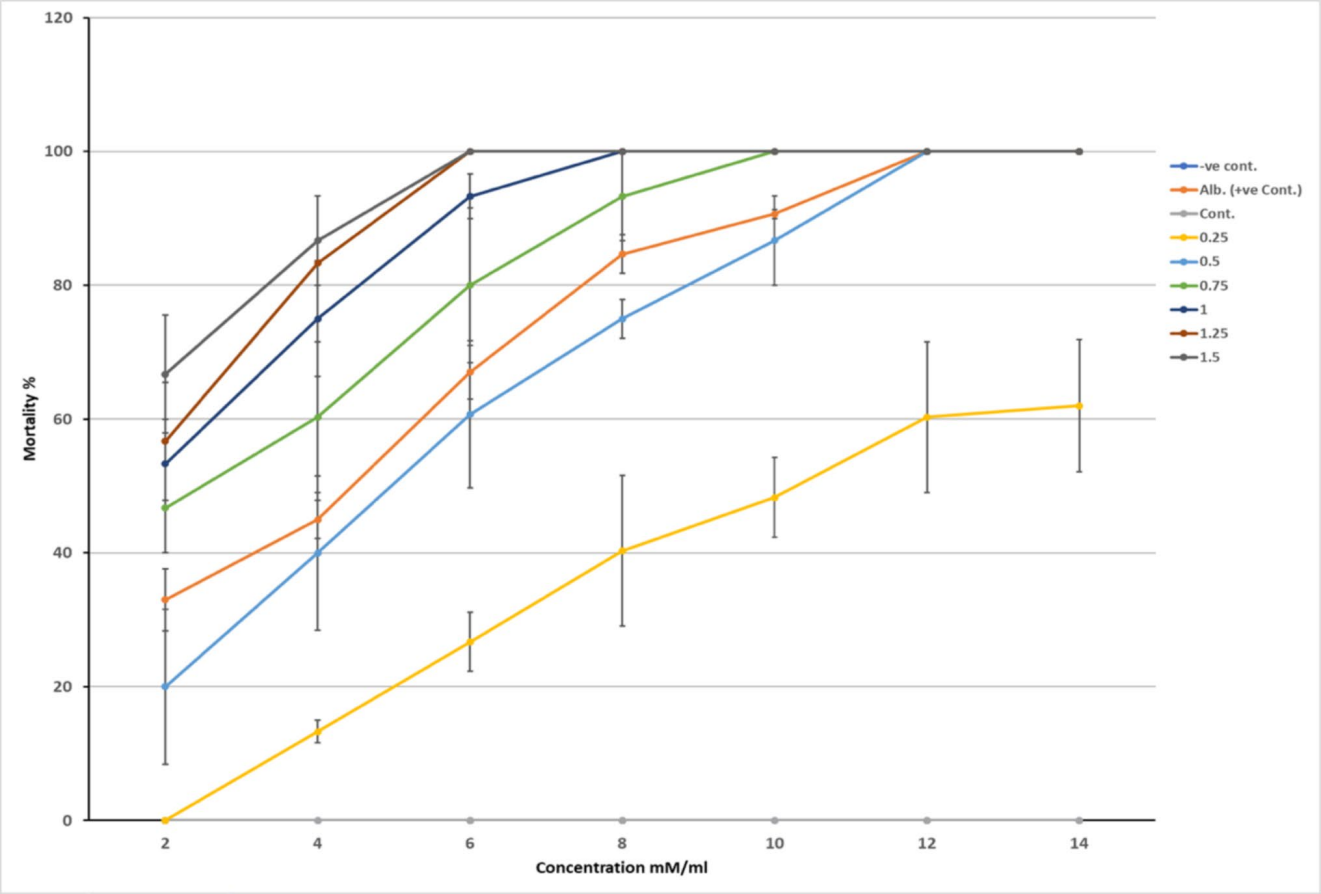
Mortality rate of adult *T. canis* cultivated in different concentrations (0.25, 0.5, 0.75, 1.25, and 1.5 mM\ml) of the ethanolic extract of quercetin during seven-time period intervals, compared with both the -ve control group (RPMI), the control group (ethanol and RPMI) and the +ve control group (albendazole)

All the cultivated worms in albendazole with ethanol (+ve control), and all concentrations (0.25, 0.5, 0.75, 1.25, and 1.5 mM\ml) of the ethanolic extract went through a phase of paralysis once they were cultivated. Due to the large size of the worms and their extremely sluggish motion, this phase was scarcely noticeable. To distinguish between the dead and paralysed cultivated worms during each exposure period, we had to immerse the worms in a phosphate-buffered saline solution for a few minutes, assess their mobility using the mechanical shock, and return them back to the cultivation medium to process them for the following exposure period.

The lowest effective dose of quercetin was 0.5 mM\ml, where 40% of the worms were dead at 4 hours, and by 8 hours, 86% had died. Moreover, quercetin at a dosage of 1 mM\ml resulted in 53% mortality after two hours of exposure and 100% mortality within 8 hours of therapy. Worms exposed to albendazole showed early mortality that started at 2 hours (33%) and gradually ascended to 90.67 at 10 hours. After the same duration of time, albendazole caused deaths twice as frequently as quercetin at a relatively similar concentration (0.25 mM). However, the moderate examined concentrations of quercetin (0.75 and 1 mM) revealed substantially higher mortalities than albendazole, especially after 4 hours of exposure.

After 8 hours of quercetin exposure, we have measured the LC50 value of 0.275 mM (95% lower confidence limit -0.767, 95% higher confidence limit 2.1) and the LC90 value of 0.55 mM (95% lower confidence limit 2.899, 95% higher confidence limit 5.767) for the worm mortality.

Two-way ANOVA test revealed a highly significant interaction (F (5, 17) = 27, *p* <0.000) between the quercetin concentrations and the worm mortality at all exposure times, confirming the in vitro dose-response effect of quercetin on the viability of the adult *T. canis*. High concentrations of quercetin (1.25 and 1.5 mM/mL) recorded a highly significant relationship with the death rate of cultivated worms in all exposure times (F (6, 20) = 7.250, *p*<0.001 and F (6, 20) = 8.667, *p*<0.000, respectively).

## Ultrastructural Damage Inflicted by Quercetin (Fig. 2)

**Fig. 2:**
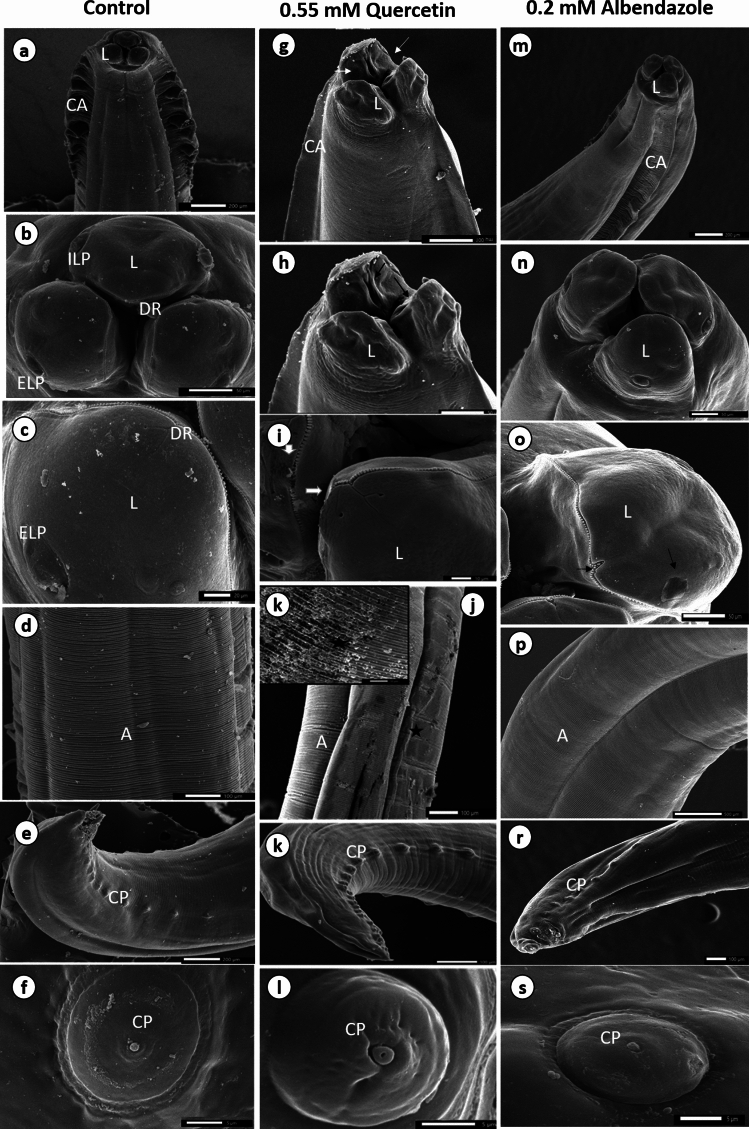
Scanning electron micrographs of male *T. canis*. a-f control group, g-m LC90 of quercetin treated group, n-s LC90 of albendazole treated group. A annulations, CA cervical alae, CP cloacal papillae, DR dentigerous ridges, ELP external labial papillae, ILP internal labial papillae, L lips. Note the expansion of the three lips to the exterior (white arrows), longitudinal furrow with fringed cuticular thickening in the internal surface of each lip (black arrow), the loosening of some tiny dentigerous ridges on the outer edge of the lips' interior surface (arrowhead), the irregularity of cuticular annulations with erosion of the cortical layer (*) and shrinking and swallowing up of caudal papillae in the quercetin-treated group. Albendazole treated worms show little flattening of labial papillae with small scars on the internal lip edge (black arrow), the blurring of cuticular transverse annulations, the shrinking of caudal papillae with a slightly swollen surface

SEM analysis revealed that adult male worms cultured in RPMI-1640 and ethanol for eight hours had a regular cylindrical shape and consistent architecture with no surface changes. These features included three well-defined, triangular, swollen lips, along with longitudinal furrows in the inner surface of each lip, fine dentigerous ridges lining the outer edge of the inner lip surface, external and internal labial papillae, a narrow, wrinkled cervical alae, a body with distinct annulations, and a curved posterior end with cloacal papillae, a button-like structure with a swollen edge.

Conversely, all worms treated with a sub-lethal dose (LC90) of quercetin for the same exposure time exhibited alterations in the overall topography, such as a reduction in size throughout their length and a shrinkage of their body wall accompanied by a thicker cuticle. The lips have expanded and opened obliquely on the exterior, exposing the internal surface of each lip's longitudinal furrow with fringed cuticular thickening. Additionally, some of the tiny dentigerous ridges that line the outer edge of the lips' interior surface have been loosening. Along the length of the body, there is an irregularity in the cuticular annulations with erosion of the cortical layer in many areas. The caudal papillae shrank in diameter and swelled up to three times more than normal. After 8 hours of incubation with 0.2 mM/ml of albendazole, the lips showed relatively little disruption, where ome small scars were detected over the internal lip edge. Labial papillae became flattened but could be distinguished. The distinctions between the cuticular transverse annulations grew blurred. The caudal papillae were barely shrunken with a slightly swollen surface.

## Histopathological Investigations Following Quercetin Treatment (Fig. 3)

**Fig. 3:**
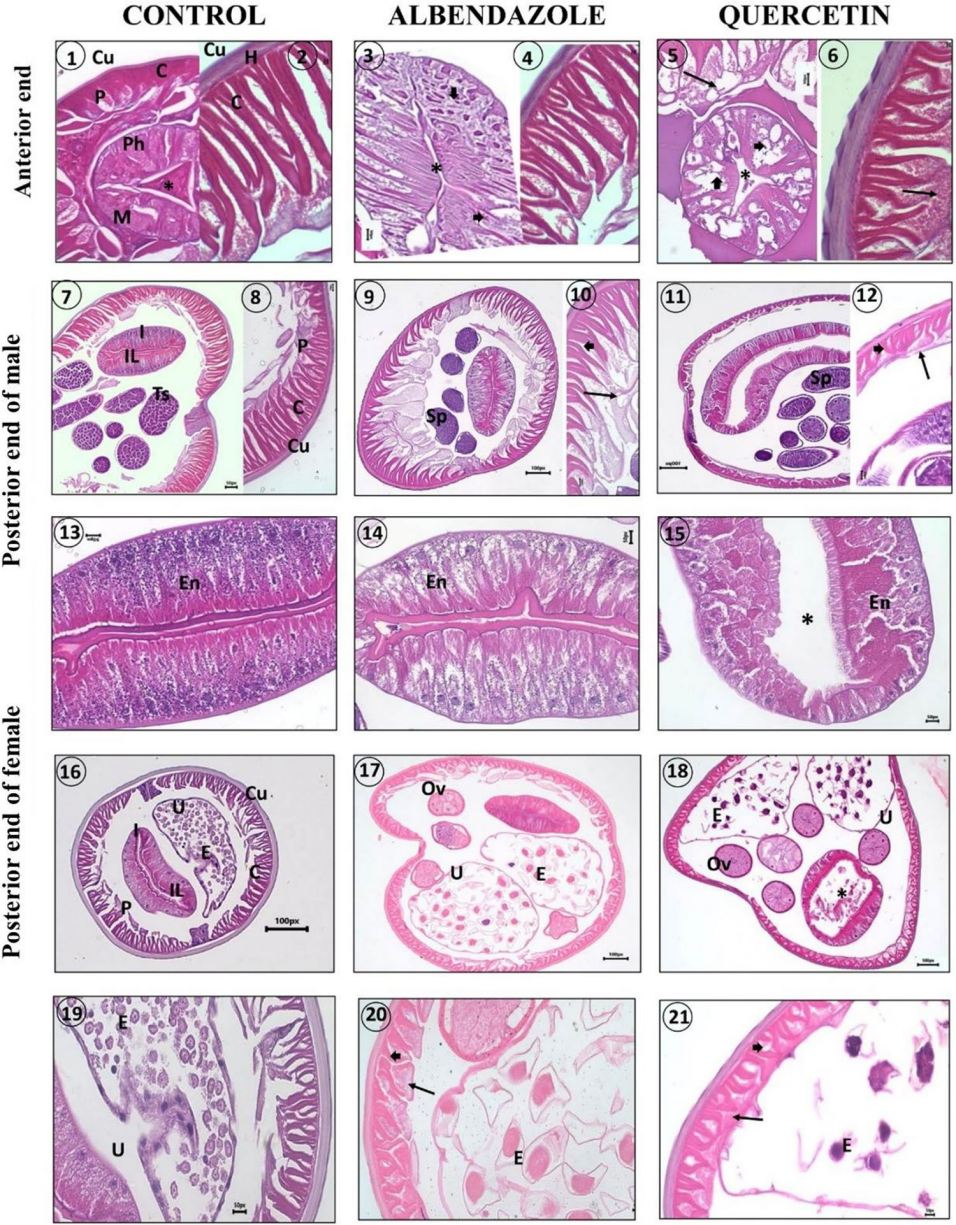
Histopathological changes in adult *T. canis* after the treatment with LC90 of quercetin compared wit worms in albendazole-treated group and control group. Figures 1–6 transverse sections in the anterior end. C. contractile part of muscle cell, Cu. cuticle, H. hypodermis, M. muscular layers of the pharynx, P. protoplasmic part of the muscle cell, Ph. pharynx. Note: the disorganization in the pharyngeal muscle layers (arrowheads) and the erosion of the pharynx's cuticular lining (*) in the albendazole group, and enlargement and well-granulation protoplasmic part of muscle cells (arrows), disorganization and vacuolation of the pharyngeal muscular fibers (arrowheads) and erosion of the pharynx's cuticular lining (*) in quercetin-treated group. Figures 7–12 transverse section in the posterior end of male. I. intestine, IL. intestinal lumen, Sp. spermatogonia, Ts. testes. Note: A marked disintegration of the contractile part (arrowhead) and enlargement of the protoplasmic part (arrow) of the muscle cells in the albendazole group, a disintegration of the contractile part (arrowhead) and granulation of the protoplasmic part (arrow) of the muscle cells in quercetin group and vacuolation in the cytoplasm of the more acidophilic spermatogonia in both groups. Figures 13–15 transverse sections in the intestinal epithelium. En. enterocytes. Note: the less granulated enterocytes in albendazole group, dilation in the intestinal lumen (*) and diffusion and contraction of more granulated enterocytes in quercetin group. Figures 16–21 transverse in the posterior region of females. E. egg, Ov. ovary, U. uterus. Note: disintegration of the contractile part (arrowhead) and granulation of the protoplasmic part (arrow) of the muscle cells in both albendazole and quercetin groups

A typical *T. canis* histology shows multiple, concentric proteinaceous cuticular layers that surround the body, and a syncytial hypodermis lies beneath the cuticle. The muscular layer consists of two distinct parts: a lengthwise, contractile fibrillar portion and a granular, non-contractile protoplasmic section that extends towards the body's center. The pharynx consists of alternatively— arranged radial and marginal muscle fibers with slightly oblique orientation, and extension between the basement membrane and the triradiate-luminal cuticle. The intestinal epithelium (enterocytes) has a well differentiated brush border which surrounds a narrow lumen. In the male, sections in different testicular regions show different developmental stages of spermatogonia, which are surrounded by marked epithelial and glandular layers. In females, two ovaries are the primary site of formation for oogonia, and the mature polyhedral eggs are stored in the uteri, which are coated with a layer of tough muscle.

In the albendazole-treated group, the most distinct changes are the disorganization of the body wall and the reduction of the layer's thickness, with evident disruptions at the underlying syncytial hypodermis and removal of the associated muscle layer. Marked disintegration of the contractile part and enlargement of the protoplasmic part of the muscle cells can be detected. Anteriorly, there is disorganization in the pharyngeal muscle layers, and the pharynx's cuticular lining is eroding. Sections in the testes show vacuolation in the cytoplasm of the more acidophilic spermatogonia. In treated females, there is a notable destruction of eggs with a reduction of their number in the uterus with a less differentiated uterine matrix. No marked changes are detected in the intestinal epithelium in both sexes, but a reduction of the glycogen content may be observed.

Reduction of the body wall thickness and swelling of the cuticular layers are marked alternations in the quercetin-treated worms. There is a distinct reduction in the length, a partial fusion and disorientation of the contractile portions of the muscle cells, while the protoplasmic portions become enlarged, extended and well-granulated. A noticeable disorganization and vacuolation are detected in the pharyngeal muscular fibers with erosion of the pharynx’s cuticular lining. In both sexes, there is a marked dilation in the intestinal lumen resulted from the rupture of the intestinal wall and the reduction of the intestinal brush border. Additionally, the enterocytes appear diffused, contracted and enclosed in denser and granulated cytoplasm, particularly at the base. Treated males show similar changes to those in the albendazole-treated group, where the cytoplasm of spermatogonia appears denser and vacuolated. On the other hand, females show observable malformations in the uterus, including egg disfiguring with a decrease in number and uterine matrix degeneration. The egg membranes were distinctively distorted throughout.

## Alterations in the Activity of Antioxidant Enzymes Following Quercetin Treatment (Fig. 4)

**Fig. 4:**
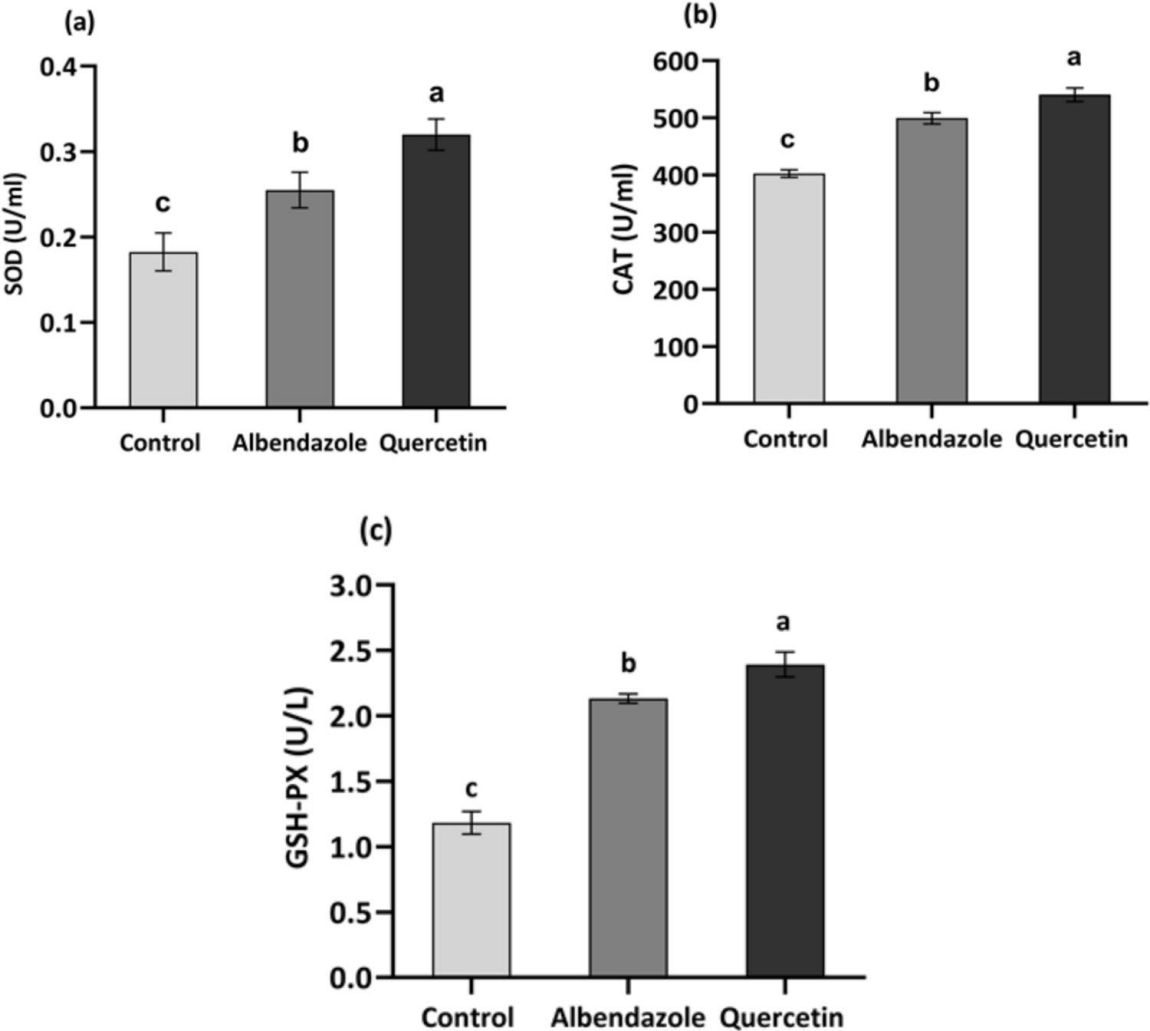
Alterations in the activity levels of oxidative stress responsive enzymes in adult *T. canis* after 8 hours of exposure time to LC90 of quercetin and albendazole compared with the control group. Groups with different letters are significantly different from each other (*p* < 0.05)

The study assessed alterations in the activity levels of oxidative enzymes involved in reducing oxidative stress in the worm's body, such as catalase (CAT), superoxide dismutase (SOD), and glutathione peroxidase (GPx), after treating the worm with LC90 of quercetin, compared with albendazole-treated and control groups. Following an eight-hour exposure time, the differences between the three groups are statistically significant for all enzymes, as indicated by the *p*-values (< 0.001 for SOD, CAT, and GSH-Px). The Quercetin-treated group shows the highest enzymatic activities for SOD, CAT, and GSH-Px, indicating strong antioxidant properties. On the other hand, the albendazole-treated group exhibited moderate enzymatic activities across all three enzymes; both groups differed significantly from the control group. The most altered activity level was observed for SOD, followed by GSH-Px.

## Discussion

Flavonoids, such as quercetin, have been the primary approach used to screen plant secondary metabolites for activity against adult nematodes in the search for substitute antihelminthics. These flavonoids have multiple mechanisms of action and low host toxicity, which have been reported to modulate pathologically altered processes during infections [35]. While screening for these new alternatives with *in vitro* assays is more expensive and time-consuming, it allows us to target different biological stages of the most health-impacted nematodes. As a follow-up to our earlier study on the nematocidal effect of quercetin on third stage larvae of *Toxocara canis in vitro* [10], the present investigation evaluates the effect of alcoholic extracts of quercetin against the adult stage.

Both quercetin and ABZ-incubated worms in the current study exhibited a slower rate of activity than the untreated controls, with quercetin's effect extending over time and albendazole's influence being immediate. As has been seen for other medications, this reduce in motility may be adequate in vivo to drive the worms out of the host's intestine [27]. In accordance with the findings of Goel *et al*. [13] and Elmahy *et al*. [10], the current study indicates that the quercetin concentration and exposure time are crucial factors that have a substantial impact on the mortality rate of the *in vitro* cultivated nematodes. The present data shows that the lowest effective dose of quercetin (0.5 mM/ml) led to 40% of the worm’s death at four hours of exposure, increased to 86% at eight hours, and 1 mM\ml resulted in 53% mortality after two hours, increasing to 100% mortality within 8 hours of therapy. The present alteration of the glycoprotein on the cuticle following the quercetin treatment might result from the fact that tannins and polyphenols selectively bind to the glycoprotein and thereby cause death [31].

The large size and sluggish mobility of the cultivated adult *Toxocara* contribute to its unapparent paralytic phase following the *in vitro* treatment with quercetin, this issue has been fixed by refreshing them in a buffered-saline solution and encouraging them to show signs of activity with a mechanical stimulus. The paralytic phase is most likely caused by the percentage of alkaloids enclosed in quercetin, which has been previously found to have an inhibitory effect on the earthworm's central nervous system [17].

Prolonged exposure to quercetin was found to be associated with the generation of a significant amount of reactive oxygen species (ROS), such as free radicals, in the nerve ring, ventral cord, and commissural connections in the adult nematodes, inducing oxidative stress in the nervous system and leading to paralysis [13]. Antioxidant enzymes like glutathione peroxidase (GSH-Px), catalase (CAT), and superoxide dismutase (SOD) not only play a vital but essential part in biological systems' ability to fend off attacks from free radicals. Superoxide dismutase (SOD) catalytically converts the superoxide radical (O2) or singlet oxygen radical (^1^O_2_) produced in tissues by metabolism or cell processes into hydrogen peroxide (H2O2) and molecular oxygen (O2). Catalase (CAT) that is prevalent in peroxisomes, prevents the formation of harmful hydroxyl radical (•HO) by converting H2O2 into water and molecular oxygen. However, because the mitochondria lack catalase, glutathione peroxidase (GSH-Px) is responsible for reducing H2O2 to water and lipid peroxides to their respective alcohols [16, 37].

In the present study, eight hours of exposure to the LC90 of quercetin duplicated the levels of oxidative enzymes more than the control worms, particularly GSH, which may help to explain quercetin's toxicity mechanism on the cultivated worms. It is possible that the burden of oxidative stress by quercetin may lead to parasite apoptosis [39] or cause adverse and irreversible modifications of the cellular macromolecular machinery in the nervous nerve cells and eventual death of the parasite [13, 24]. In addition, the indirect biological effects of quercetin could have positive antiparasitic effects, due to the induction of microbicidal responses, such as the production of nitric oxide and some cytokines [39].

It has been demonstrated that anthelmintic drugs can infiltrate target parasites through the mouth or by diffusing through their surface [44]. Thus, destroying the nematode's surface through trans-cuticular passive diffusion was one of the main impacts of any anthelmintic [23]. In the present work, we demonstrated for the first time the physical changes inflicted on the adult *Toxocara* following quercetin treatment. The data revealed that quercetin disrupted both ends and other body regions, including the destruction of the pharyngeal cuticular lining. The thickening of the cuticle, swelling of the caudal papillae, and deformation of the surface cuticular annulations of the cultivated worms after quercetin treatment may be related to the presence of isoflavonoids groups that alter the phosphatase enzyme in the tegument of helminths [20] and uncouple the oxidative phosphorylation that interferes with the glycoprotein on the cuticle and causes parasite death [17]. Furthermore, the entire body's shrinkage and the peculiar opening of the mouth with some dentigerous ridges of the lips falling out could be related to the enlargement and disorientation of the protoplasmic portion and the detachment of the fibrillar portion of the muscle bundles that have been detected in the anterior body of the cultivated worms. Albendazole therapy also causes physical damage, such as partial body shrinkage and disturbance of the cuticle, but less damage than quercetin.

Less stressful alternations have been detected in the third-stage larvae of *T. canis* after the same exposure time to quercetin [10], the damage was concluded by the appearance of wrinkles and cracks and a lack of cuticle folds. Cuticle seems to be a target structure easily accessible to macromolecules like quercetin due to its high content in proline-rich collagens, that could affect several functions of the cuticle such as permeability [41], molting [21], locomotion [32], response to the environmental stress [9] and immune response [50].

In the present study, we demonstrated the histological constitutions of *T. canis* caused by quercetin in the body wall, pharyngeal lining, intestinal epithelial cells, uterine chamber and growth zone of the testis compared with albendazole-treated and control worms. Whereas, albendazole treatment, partly reduced the body wall layer's thickness, disrupted the underlying syncytial hypodermis, eroded the pharynx's cuticular lining and destructed the egg in the uterine chamber, quercetin at 0.55 mM drastically altered these structures with a swelling of the cuticular layers, reduction and disorientation of contractile portions of the muscular layer and enlargement of the protoplasmic portion, vacuolation in the pharyngeal muscular layer, diffusion and granulation of the intestinal epithelium, egg disfiguring and vacuolation of the cytoplasm spermatogonia.

It is possible to conclude that quercetin has a more extensive and varied effect on tissues, but this effect necessitates prolonged exposure. In contrast, albendazole has a more focused and severe effect. The main antiparasitic mechanism of quercetin may underlay many inhibitory effects including mitochondrial dysfunction, reduced expression of heat shock proteins, inhibition of specific enzymes involved in fatty acid synthesis and the glycolytic pathways, and impairment in iron absorption [28].

## Conclusion

In the present study, quercetin caused marked alterations in the body wall of *T. canis* accompanied by noticeable disorganization of both the pharyngeal muscular layer and intestinal wall, as well as malformation of the uterus. This impact may be associated with the generation of a significant amount of reactive oxygen species (ROS), which duplicated the levels of the three oxidative enzymes, particularly GSH in the treated worms. In addition to its ability to combat viral and protozoal infections, quercetin presented itself as a drug that might become important in nematode chemotherapy. Due to the poor absorption of flavonoids, particularly quercetin aglycone in the mammalian intestine [38, 43], the *in vivo* application of quercetin requires a comprehensive structural study, synthesis of chemicals and recombinants, identification of molecular targets, therapeutic optimization, and effective delivery methods. Furthermore, grasping the balance between quercetin’s antioxidant and potential pro-oxidant effects could facilitate more targeted applications of quercetin in both the prevention and treatment of parasitic diseases. However, further studies are required to establish the anthelmintic potential of quercetin in combination with the commonly used anthelmintic drugs under *in vivo* conditions.

## Supplementary Information

Below is the link to the electronic supplementary material.

Supplementary file1 (DOCX 16 kb)

## Data Availability

No datasets were generated or analysed during the current study.
