# Supplementary material for: In vitro Evaluation of the Nematicidal Efficacy of Quercetin on Adult Toxocara canis
Source: Acta Parasitol. 2025 Apr 23;70(3):96. doi: 10.1007/s11686-025-01026-x (PMC12018618; doi:10.1007/s11686-025-01026-x)
Supplement: Supplementary file 1 — Supplementary file1 (DOCX 16 kb) [file 11686_2025_1026_MOESM1_ESM.docx]

**This table shows the mortality rate of adult *T. canis* cultivated in different concentrations (0.25, 0.5, 0.75, 1.25, and 1.5 mM\ml) of the ethanolic extract of during seven-time period intervals, compared with both the -ve control group (RPMI), the control group (ethanol and RPMI) and the +ve control group (albendazole).**

| **Groups** | **-ve control (RPMI)** | **+ve control (Alb.)** | **Control (ethanol and RPMI)** | **Quercetin concentrations** | | | | | |
| --- | --- | --- | --- | --- | --- | --- | --- | --- | --- |
| **hours** |  |  |  | **0.25** | **0.5** | **0.75** | **1** | **1.25** | **1.5** |
| **2h** | **0** | **33±8** | **0** | **0** | **20±11** | **46.7±6.67** | **53.3±11.5** | **56.7±15.3** | **66.7±15.3** |
| **4h** | **0** | **45±5** | **0** | **13.3±2.89** | **40±20** | **60.3±11.26** | **75±15** | **83.3±5.8** | **86.7±11.5** |
| **6h** | **0** | **67±7** | **0** | **26.7±7.64** | **60.7±19** | **80±20** | **93.3±5.8** | **100±0** | **100±0** |
| **8h** | **0** | **84.67±5.03** | **0** | **40.3±19.50** | **75±5** | **93.3±11.5** | **100±0** | **100±0** | **100±0** |
| **10h** | **0** | **90.67±1.15** | **0** | **48.3±10.40** | **86.7±11.55** | **100±0** | **100±0** | **100±0** | **100±0** |
| **12h** | **0** | **100±0** | **0** | **60.3±19.50** | **100±0** | **100±0** | **100±0** | **100±0** | **100±0** |
| **14h** | **0** | **100±0** | **0** | **62±17.09** | **100±0** | **100±0** | **100±0** | **100±0** | **100±0** |
